# Digital Transformation on Enterprise Green Innovation: Effect and Transmission Mechanism

**DOI:** 10.3390/ijerph191710614

**Published:** 2022-08-25

**Authors:** Hua Feng, Fengyan Wang, Guomin Song, Lanlan Liu

**Affiliations:** 1 School of Accounting, Shandong Women’s University, Jinan 250300, China; 2 School of Public Policy & Management, China University of Mining and Technology, Xuzhou 221116, China; 3 Postgraduate Studies Unit, College of Business, Universiti Utara Malaysia, Sintok 06010, Malaysia

**Keywords:** digital transformation, green innovation, R&D investment, government subsidies, income tax burden

## Abstract

With the development of blockchain, big data, cloud computing and other new technologies, how to achieve innovative development and green sustainable development in digital transformation has become one of the key issues for enterprises to obtain and maintain core competitiveness. However, little of the literature has paid attention to the impact of digital transformation on enterprise green innovation. Using the data of Chinese A-share listed companies from 2010 to 2020, this paper empirically analyzes the impact of enterprise digital transformation on green innovation and its transmission mechanism, by constructing double fixed-effect models. The results show that digital transformation has remarkably promoted the green innovation of enterprises. R&D investment, government subsidies, and income tax burden have played a conductive role between digital transformation and enterprise green innovation. Furthermore, digital transformation can significantly promote the high-quality green innovation of enterprises and also plays a more significant role in promoting the green innovation of high-tech enterprises and state-owned enterprises. A robustness test is carried out by using the lag data and changing the measurement methods of the dependent variable and independent variables, and the research conclusions are still valid. Based on resource-based theory and dynamic capability theory, this paper reveals the impact path of digital transformation on enterprise green innovation, further expanding the research field of digital transformation and enriching the research on the influencing factors of enterprise green innovation. This paper provides policy suggestions for the government to improve the enterprise green innovation level by increasing government subsidies and providing tax incentives and also provides reference for digital transformation enterprises to accelerate green innovation by increasing R&D investment, obtaining government subsidies, and acquiring tax policy support.

## 1. Introduction

With the development of blockchain, big data, cloud computing, and other new technologies, digital technologies have brought profound changes to the socioeconomic field. Digital transformation has become a crucial strategic decision in the modern management of enterprises and the transformation of information systems [1,2], which has attracted extensive attention from the international community [3] and also has made the research on enterprise digital transformation become one of the hot issues in the academic community. In the Outline of the 14th Five-Year Plan, the Chinese government proposed to drive the reform of the production mode and the lifestyle and governance mode with digital transformation. In 2020, the National Development and Reform Commission of China put forward the initiative of “the digital transformation of partnership”. In 2022, China’s Government Work Report proposed that digital development should be accelerated, new advantages of the digital economy should be created, and digital industrialization and industrial digital transformation should be promoted together, which shows that digital transformation has risen from the enterprise level to national strategy [4].

As an innovative paradigm integrating the concepts of ecological environment protection and sustainable development, green innovation is mainly manifested in the design and development of new processes, new technologies, and new products conducive to energy conservation, emission reduction, and environmental protection [5,6]. With the deep penetration of the concept of sustainable development, green innovation also emphasizes the renewable design and environmental-protection process management of the whole innovation industry chain [7,8]. Under the global low-carbon development strategy, green innovation has been regarded as a key factor to coordinate economic growth and green low-carbon development and an essential path to achieve regional economic sustainable development [9].

**Figure 1:**
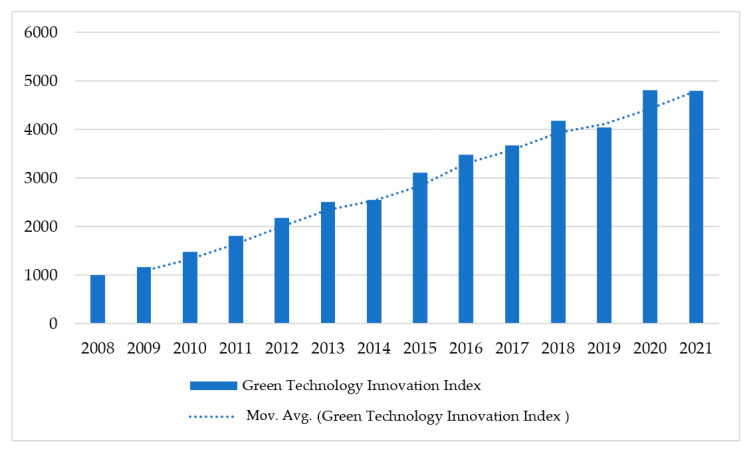
Green technology innovation index of Chinese enterprises. Note: Mov. Avg. represents the moving average of the green technology innovation index. Data source: Zero One Think Tank, https://www.01caijing.com (accessed on 5 July 2022).

In recent years, promoting green low-carbon development and realizing the comprehensive green transformation of economic and social development are momentous strategic layouts for China to promote high-quality economic and social development, and there are also lasting economic and social changes [10]. Since 2008, the green innovation of Chinese enterprises has maintained a rapid growth rate. According to the statistics of Zero One Think Tank, which is a famous decision-service institution in China, the green technology innovation index of Chinese enterprises has increased from 1000 in 2008 to 4791.2 in 2021 (Figure 1). The compound annual growth rate reached 11.84%. The number of green patent applications of Chinese enterprises has increased from more than 43,000 in 2008 to more than 150,000 in 2021, while the number of green patents authorized increased from 19,000 in 2008 to 184,000 in 2021, which verifies that the green technology innovation of Chinese enterprises has been growing rapidly.

Practice has proven that promoting green innovation of enterprises is the key task of establishing and improving the green low-carbon circular development economic system, while digital transformation is considered to have great potential in improving operations and stimulating growth [11]. Therefore, it is very imperative to discuss how digital transformation affects enterprises’ green innovative products and services [12] and how enterprises realize green innovative development in perceiving and responding to digital transformation [13]. In other words, in the era of the digital economy, enterprise digital transformation is no longer a simple problem of technological upgrading, as it has gradually become a strategic choice that affects the green and sustainable development of enterprises [14].

Existing studies have discussed the connotation of digital transformation [15,16], the economic consequences [17,18], and the influencing factors of enterprise green innovation [19,20] and obtained rich research conclusions. However, the impact of digital transformation on enterprise green innovation and the transmission mechanism of digital transformation on green innovation still lack of corresponding exploration. Therefore, different from the current research on digital transformation, this paper actively explores the following questions. (1) What impact will digital transformation have on enterprise green innovation? (2) What is the potential transmission mechanism? (3) Will this impact affect the quality of green innovation? (4) Is there industrial heterogeneity in this impact? The answers to the above questions will help us to accurately understand the role of the digital economy and the digital transformation strategy being promoted for enterprise green innovation, to then help government departments formulate targeted policies to promote the digital transformation and enterprise green innovation.

In order to deeply explore the relationship between digital transformation and enterprise green innovation and the transmission mechanism, this paper uses the data of Chinese A-share listed companies from 2010 to 2020 to empirically test the impact of digital transformation on enterprise green innovation, by building a time and industry dual fixed-effect model. It is found that the implementation of digital transformation has significantly improved enterprise green innovation. From the perspective of the transmission mechanism, R&D investment, government subsidies, and income tax burden have played a mediating effect between digital transformation and enterprise green innovation. In addition, the digital transformation of different property rights and industries shows different sensitivities to the response of enterprise green innovation, which is specifically manifested in that digital transformation has a more significant impact on high-quality green innovation, and the attributes of high-tech enterprises and state-owned enterprises can significantly improve the effect of digital transformation on green innovation. The robustness test carried out by changing variables, the lag test, and other methods still supports these conclusions. From the perspective of China’s current digital economy strategy and the practice of enterprise digital transformation, improving the vitality and quality of enterprise green innovation is a crucial measure to promote sustainable economic development and green transformation development. The conclusions of this paper will provide forceful empirical support and experience reference for the development of the digital economy and the process of enterprise digital transformation.

Based on the above analyses, the main contributions of this paper include three aspects. First, existing studies have mainly explored the micro impact of digital transformation on enterprises from the perspective of business digitalization [15,21] and strategic digitalization [16], but they have rarely discussed how digital transformation affects enterprise green innovation. Therefore, this paper deeply analyzes how digital transformation affects enterprise green innovation, expands the research field of the economic consequences of digital transformation, reveals the transmission path of digital transformation on enterprise green innovation from the perspectives of dynamic capability theory and resource-based theory, and further expands the research field of the economic consequences of digital transformation. Second, scholars have mainly analyzed the impact of external factors represented as market driven [22,23] and system driven [24,25], as well as internal factors represented by green orientation [26,27] and green-technology capability [28,29] on enterprise green innovation. Nevertheless, less attention has been paid to the research on digital transformation. From the perspective of digital transformation, this paper discusses its impact on green innovation, and enriches the research scope of the influencing factors of enterprise green innovation. Third, a few scholars have studied the impact of digital technologies on green innovation, but these studies are relatively scattered, mainly from single factors such as digitalization [30], blockchain [31], and big data [32], so there is a lack of systematic research on the relationship between digital transformation and green innovation from an overall perspective. This paper comprehensively studies the impact of the overall digital transformation on enterprise green innovation, and makes a heterogeneity analysis from the perspective of green innovation quality, property rights nature, and industry, which further expands the research on the impact of digital transformation on green innovation.

The main structure of this paper is as follows: Section 2 is the literature review, Section 3 puts forward research assumptions, Section 4 explains the sample sources, variables definition, and empirical models, Section 5 carries out empirical analysis, Section 6 is discussion, Section 7 summarizes the research conclusions, and finally, Section 8 puts forward recommendations on how to improve enterprise green innovation from the perspective of digital transformation.

## 2. Literature Review

### 2.1. Research on Digital Transformation

Digital technologies play an increasingly critical role in promoting global economic development, which has affected society, enterprises, and people’s lives [33]. These technologies include, but are not limited to, big data, artificial intelligence, cloud computing, blockchain, and the Internet of things [34]. From a global perspective, digital transformation has triggered the transformation of new business models and has created and acquired enterprise value based on new logic and ideas [35,36], which has become one of the key factors to subvert and reshape business models [21].

Generally speaking, digital transformation can be divided into business digital transformation and strategic digital transformation [15]. Business digital transformation tends to emphasize the application of digital technologies in processes and systems and to refine and expand the business process at the business level, so as to realize the digitalization of organizational operations [37,38]. Compared with business digital transformation, strategic digital transformation emphasizes the implementation of more profound changes within the enterprise to achieve value creation, including changes in business processes, business models, and organizational culture [16]. Strategic digital transformation is also divided into digital business strategy (DBS) and digital transformation strategy (DTS). DBS, which not only helps reshape enterprise strategy but also can realize value creation [11,39], is considered to be a forward-looking overall strategy, believing that digital transformation can fundamentally change enterprise strategies, business processes, products, and services [40] as well as overturn the status and role of participants in the industry [41]. However, DBS indicates the general scheme of enterprise digital transformation [1,42] but does not specify how to achieve these goals, and DTS solves this problem from a practical level. DTS includes applying digital technologies to promote organizational cooperation [43], improving organizational flexibility [44,45] and updating corporate culture to improve performance [46,47], which provides practical guidance for enterprise digital transformation [48].

In practice, digital transformation has been proven to have a far-reaching impact on enterprise activities. First, digital transformation can effectively reduce information communication and transaction costs [49], improve the availability and transparency of market information [50], and enable enterprise management to timely perceive opportunities and challenges in the business process [42], thus improving the financial performance of enterprises [17,18,51]. Second, digital transformation can also provide enterprises with specific advantages in international competition [34,52], such as changing corporate governance structure, business processes, and business models, and improve managers’ business perception and decision-making ability, so that enterprises can obtain competitive advantages in an uncertain economic environment [53,54]. Third, digital transformation can enhance the ability of enterprises to cope with changes in the external environment and form and maintain the dynamic ability of sustainable development of enterprises [55,56], thus helping to improve enterprise innovation [57]. In recent studies, scholars also tested the impact of economic and technological transformation (by using economic complexity) on energy use and environmental degradation [58,59], which further enriched the research on the economic effects of digital transformation.

### 2.2. Research on Green Innovation

Green innovation is generally regarded as an innovation paradigm that considers ecological environmental protection and sustainable development, also known as ecological innovation or environmental technology innovation [60], which is specifically manifested in new processes, new technologies, and new products of enterprises in energy conservation, emission reduction, pollution control, green services, and other aspects [5,6]. Some scholars believe that green innovation refers to the use of environmentally friendly raw materials in the manufacturing or design process, to develop sustainable products and services [7,8]. Therefore, green innovation is regarded as an innovation process throughout the whole business cycle, which means that the design, production, and sales of green products should reflect the sustainable protection of the environment [61].

Scholars mainly discussed the influencing factors of green innovation from the perspective of external and internal factors [62,63]. The external factors mainly include two perspectives, system-driven and market-driven. In the view of system driven, the most typical is the proposal of the “Porter Hypothesis” [24]. The “Porter Hypothesis” puts forward that environmental regulation is a vital innovation-driving factor, and reasonable environmental regulation will promote innovation to improve enterprise profitability [25,64]. A large number of studies have been carried out by many scholars to verify this view. For instance, the research of Larrán Jorge et al. (2015) and Zhang et al. (2020) confirmed that strict environmental regulation can not only fight pollution but also urge enterprises to improve green innovation [65,66]. However, some scholars believe that environmental regulation will increase the economic cost of enterprises to fulfill their environmental responsibilities, which will inhibit enterprise green innovation [67,68]. In addition to the linear relationship research, a few scholars believe that environmental regulation has a nonlinear relationship with enterprise green innovation [25]. Besides environmental regulation, other institutional factors such as government subsidies are also essential factors affecting green innovation [69]. Some studies believe that government subsidies can make up for the high costs and risks brought by green innovation and promote enterprises to actively carry out green innovation [19,20]. Furthermore, green finance policy is also a crucial institutional factor driving green innovation of enterprises [70,71].

It is believed that the market driven perspective is another external factor affecting green innovation. Based on the environment–market theory, green products may also bring private benefits to stakeholders in addition to reducing the public benefits of environmental pollution [22]. For instance, besides reducing environmental pollution, products can also bring added value to customers; thus, customers will be more willing to pay a higher price to buy green products. Therefore, the demands of customers, suppliers, and other stakeholders also significantly affect green innovation [23,72]. At the same time, green market and green energy are also critical factors to promote enterprises to carry out green innovation [73].

In terms of internal factors, the organizational elements of enterprises, such as green orientation and green technology capability, are momentous factors affecting green innovation. Green orientation is the cognitive process of enterprises for environmental protection management, which is divided into internal green orientation and external green orientation. The former refers to the internal value recognition and ethical standards of environmental protection commitments in corporate culture, while the latter refers to the green behavior implemented by enterprise managers to meet the environmental protection needs of external stakeholders [26]. For instance, Bu et al. (2020) found that internal and external environmental orientation promoted enterprise green supply chain management, including environmental selection, monitoring, and cooperation with suppliers [27]. Another internal factor affecting green innovation behavior is green technology capability, including green resources, environmental management system (EMS), and organizational flexibility related to the implementation of green innovation by enterprises [74]. As proof, Muisyo and Qin (2021) found that the practice of green human resource management could significantly promote the green innovation performance of enterprises [28], while Khanna and Deltas (2009) found that EMS enabled enterprises to improve efficiency, thus reducing the cost of green technology innovation and helping to achieve green innovation [75].

### 2.3. Impact of Digital Technologies on Green Innovation

A few scholars have studied the impact of digital technologies on green innovation, but these studies are relatively dispersed, mainly in terms of digitalization, blockchain, Internet, and big data. First, existing studies believe that digital technologies can alleviate the problem of enterprise information asymmetry, improve the level of information sharing [53], push the integration of R&D resources and knowledge, help to enhance the innovation vitality of enterprises [30], optimize the allocation of green R&D resources, and, thus, promote green innovation of enterprises [76]. For instance, Li and Shen (2021) found that digitalization could improve the level of green innovation, especially in the case of imperfect internal control and institutional ownership [76]. Using the survey data of 215 enterprises, El-Kassar and Singh (2019) found that digitalization positively affected enterprise green innovation activities, thereby affecting enterprises’ competitive advantages [77]. Second, blockchain technology can shape a fair technological ecosystem through encryption algorithms, which also has an important impact on enterprise green innovation. For instance, Li (2021) explored the path of green technology innovation based on blockchain technology and believed that the application of the ipbft algorithm could better provide protection for green technology innovation [78]. Jiang et al. (2021) proposed to build an ecological innovation system of smart cities with the help of blockchain and believed that blockchain technology is conducive to achieving sustainable green innovation [31]. Third, Internet, big data, and other technologies also have a significant impact on green innovation. As proof, Wang et al. (2022) analyzed the internal mechanism of the impact of Internet development on green economic growth. The empirical results found that Internet development could promote the upgrading of industrial structure, accelerate enterprise innovation, and indirectly promote green economic growth [79]. Waqas et al. (2021) found that manufacturing enterprises could formulate optimization plans for environmental problems based on big data analysis technology, alleviate the pressure of natural resources, and promote green innovation [32].

Scholars have carried out a great deal of research from the perspective of digital transformation, green innovation, and the impact of digital technologies on green innovation, which provides the basis for this research, though there are still some deficiencies. First, scholars mainly analyze the connotation and classification of digital transformation [37,38] and the economic effect of digital transformation [17,18], while they pay less attention to the impact of digital transformation on enterprise green innovation. Second, existing studies have revealed the impact of these factors on enterprise green innovation from the perspective of internal factors [26,27] and external factors [65,66], while there is less research on digital transformation. Third, a few scholars have studied the impact of digital technologies on green innovation, whereas these studies are mainly based on single factors such as digitalization [30], blockchain [31], big data [32], and lack of systematic research on the relationship between digital transformation and green innovation from a holistic perspective. In order to solve these problems, this paper deeply analyzes how digital transformation affects enterprise green innovation, reveals the transmission path and heterogeneous impact of digital transformation on enterprise green innovation, and provides recommendations for the government to formulate differentiated and accurate policies to promote enterprise green innovation.

## 3. Theoretical Analysis and Research Assumptions

### 3.1. Digital Transformation and Green Innovation

**Figure 2:**
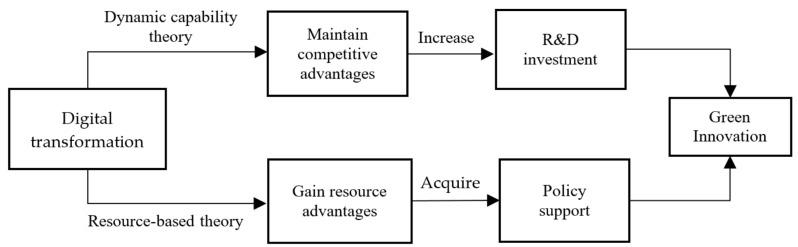
Transmission mechanism of the impact of digital transformation on green innovation.

Scholars’ views on how enterprise digital transformation affects green innovation mainly include “dynamic capability theory” and “resource-based theory” (Figure 2). The first view is that dynamic capabilities are considered to be the ability of an organization to purposefully create, expand, and adjust its competitive advantages [34], so that enterprises can adapt to the rapidly changing environment and make dynamic adjustments [55]. In the process of scale expansion, enterprises need dynamic capabilities to create and maintain competitive advantages over other enterprises [34]. It has been found that dynamic capabilities play a critical role in the digital transformation of enterprises [80]. Digital transformation is not a simple digitalization based on the original capabilities. Enterprises need to make full use of existing capabilities, while constantly exploring new capabilities in the business process discovering opportunities and challenges from the internal and external environment through perceptual capabilities [81], and maintaining dynamic innovation capabilities and competitiveness. In order to acquire and maintain dynamic capabilities, enterprise managers need to maintain keen insight and innovation awareness and increase green innovation output through increasing R&D investment [82], so as to maintain the competitive advantage of green products and services. The second view is the resource-based theory, which believes that the advantages of digital transformation can help enterprises search for more favorable information [83]. With the help of digital technologies, enterprises can accelerate the transmission speed of information in organizations and promote the sharing of information within enterprises [84]. Under the background of the continuous introduction of innovation driven and green sustainable development policies, digital transformation can help enterprises reduce the cost of environmental information collection and analysis, enable enterprises to quickly master the changes of the latest environmental policies, and better grasp the direction of the government’s environmental policies. This can help enterprises obtain more government policy support [85], ease the financing constraints of enterprises’ green R&D activities, and encourage enterprises to implement green innovation [76]. Therefore, this paper proposes the following assumption:

**Hypothesis** **1.** *Digital transformation has a critical role in promoting enterprise green innovation.*

### 3.2. Digital Transformation and Green Innovation Quality

Some studies have found that enterprises will carry out strategic innovation [86] and unilaterally pursue increasing the number of innovations [87] to meet government policies, which may lead to the reduction in R&D investment [88], thus adversely affecting the quality of innovation. Therefore, it is necessary to explore the impact of digital transformation on the quality of green innovation. In the context of digital economy, digital transformation forces enterprises to change their value creation path through digital technologies from the perspective of sustainable development strategy, so as to maintain their competitiveness in the market [18], which also provides enterprises with opportunities to catch up with peers and subvert the market [89]. Digital transformation has become a momentous way for enterprises to obtain core competitiveness [53], and the maintenance of this core competitiveness and the catch-up of new technologies require enterprises to improve the innovative technology of products and services through continuous R&D activities, which inspires enterprises to pay more attention to high-quality innovation activities [70] and focus R&D funds on high-quality innovation activities that can improve the technological level of enterprises to aggrandize high-quality innovation output [88]. Therefore, this paper proposes the following assumption:

**Hypothesis** **2.** *Digital transformation has a more significant impact on high-quality green innovation.*

### 3.3. Digital Transformation and Green Innovation of High-Tech Enterprises

High-tech enterprises, industries with R&D technological innovation as their core competitiveness, play an essential role in promoting regional economic growth. High-tech enterprises have the characteristics of high R&D investment, intensive innovation resources, advanced technology, etc. [90], which make them have natural advantages in the process of digital transformation. First, high-tech enterprises can make full use of the R&D advantages and digital technologies to empower the R&D process in the process of digital transformation, so as to improve the efficiency of green innovation activities and increase the output of green innovation. Second, high-tech enterprises have gathered a large number of high-level human resources with digital technologies. The ability of employees can effectively promote the improvement effect of digital transformation on green innovation [12], which makes high-tech enterprises obtain more green innovation output under the same external conditions. Third, high-tech enterprises have an innovative and shared organizational culture, higher organizational leadership, and industry sensitivity and can quickly respond to the changes brought about by digital transformation [91], thus having a positive impact on green innovation. Therefore, this paper proposes the following assumption:

**Hypothesis** **3.** *Digital transformation has a more significant impact on the green innovation of high-tech enterprises.*

### 3.4. Digital Transformation and Green Innovation of State-Owned Enterprises

It is considered that state-owned enterprises play a vital role in economic development, such as boosting regional economic growth [92], accelerating industrial upgrading [93], and promoting environmental improvement [94]. The government is a key stakeholder in the operation and management of state-owned enterprises [93], which makes state-owned enterprises have a special governance structure. Existing studies have shown that state-owned enterprises may have more advantages than private enterprises, when making innovation investment in special fields [95]. First, private enterprises pay more attention to the short-term return rate of innovation investment, while state-owned enterprises carry out green innovation investment in order to obtain long-term environmental benefits and achieve sustainable development, rather than requiring enterprises to immediately generate investment returns. Second, state-owned enterprises usually have a larger enterprise scale and financial strength, which makes them have a stronger ability to resist innovation risks [96]. Third, state-owned enterprises can obtain more institutional investors’ innovation capital support because of the closer relationship with the government [97], thus reducing their innovation-financing constraints, which makes their green R&D activities less affected by external environmental changes. It can be inferred that state-owned enterprises pay more attention to long-term returns in the process of digital transformation, so they have the stronger ability to bear innovation risks and the lower degree of financing constraints, compared with non-state-owned enterprises, which makes state-owned enterprises have a more significant effect in promoting green innovation. Therefore, this paper proposes the following assumption:

**Hypothesis** **4.** *The promotion effect of digital transformation on the green innovation of state-owned enterprises is more significant.*

**Figure 3:**
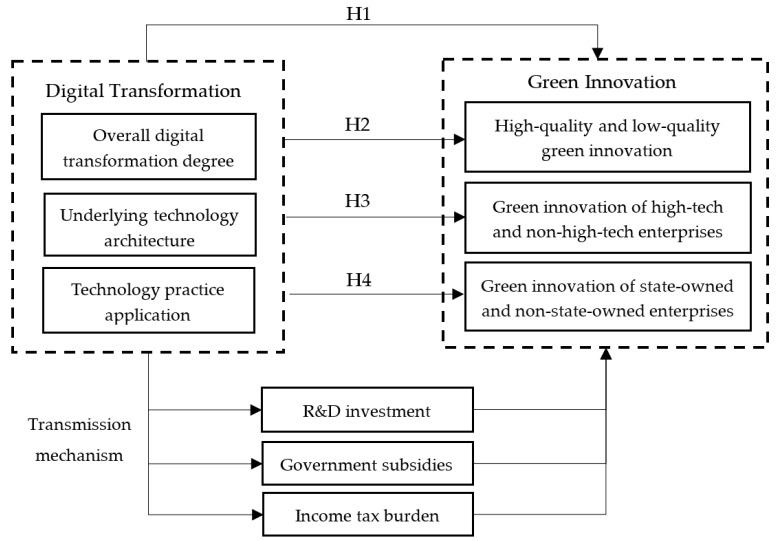
The research framework.

Based on the above analysis, the research framework of this paper is as follows (Figure 3):

## 4. Empirical Design

### 4.1. Sample Selection and Data Sources

Considering that Chinese enterprises officially started the process of digital transformation through large-scale online transactions and digital marketing in 2010, the green list of International Patent Classification (IPC) was launched in 2010, and the availability of data of other major variables, this paper takes the empirical data of Chinese A-share listed companies from 2010 to 2020 as the research sample. The main data sources include: (1) enterprise green innovation data comes from Chinese Research Data Services Platform (CNRDS, https://www.cnrds.com (accessed on 4 January 2022)); and (2) digital transformation, R&D investment, government subsidies, income tax burden, and other data are obtained from China Stock Market Accounting Research Database (CSMAR, www.gtarsc.com (accessed on 4 January 2022)). At the same time, in order to ensure the reliability of the data, companies in financial and insurance industries, ST, *ST companies, and samples with missing data are excluded. In the regression analysis, the major continuous variables are abbreviated to 1%.

According to the latest report released by Gartner, a famous global consulting and research institution, the digital economy will account for nearly 40% of China’s economy in 2021. In August 2022, according to the decision of the Joint Committee on Digital Economy Partnership Agreement (DEPA), China has formally established the working group on accession to DEPA. It has been verified that China has made outstanding achievements in the development of the digital economy, which further accelerates and stimulates the process of enterprise digital transformation. According to the statistics of the International Data Corporation (IDC), China’s industrial Internet regional platform and service market reached USD 264 million in 2021, a 28.6% increase over 2020. In 2020, China’s industrial Internet ranked second in the world, verifying that the digital transformation of Chinese enterprises has developed rapidly. Therefore, it is representative to conduct research based on data of Chinese enterprises.

### 4.2. Variables Definition

#### 4.2.1. Dependent Variables

Drawing on the research of Husnaini and Tjahjadi (2021) [98], Liao (2020) [99], and others, considering that it takes 1–2 years to obtain authorization after patent application, and patents may be put into production to create profits and during this period, the number of patent applications is more timely than the amount of authorization, so the number of green patent applications is used to measure green innovation. Moreover, given that the impact of business digital transformation on business green innovation may be lagging, next year’s data on green innovation is being used for empirical testing.

According to the provisions of China’s Patent Law, green invention patents focus on the innovation of technologies and methods in pollution discharge and environmental governance, while green utility models focus on the innovation of the shape and structure of environmental governance products, which shows that green invention patents are more effective in improving the level of enterprise green technology innovation [88]. Hence, this paper uses green invention patents to measure high-quality green innovation and uses green utility model patents to measure low-quality green innovation.

#### 4.2.2. Independent Variables

Drawing on the research of Verhoef et al. (2021) [35], Zhai et al. (2022) [18], and others, this paper uses text analysis to measure the degree of digital transformation of enterprises. The total number of texts involving artificial intelligence, big data, cloud computing, blockchain, and digital technology application is defined as the overall digital transformation degree. In order to further distinguish various indicators, the number of text occurrences of artificial intelligence, big data, cloud computing, and blockchain is defined as the underlying technology architecture, which is used to measure the degree of digital transformation of underlying technology. Moreover, the number of text occurrences of digital technology application is defined as the technology practice application, which is used to measure the degree of digital transformation at the application level. The above indicators are the natural logarithm, after adding 1 to the number of occurrences of relevant text.

#### 4.2.3. Mediating Variables

Drawing on the research of Yang et al. (2020) [100], Wang et al. (2022) [101], and other scholars, this paper uses R&D investment, government subsidies, and income tax burden as mediating variables combined with the transmission mechanism of the impact of enterprise digital transformation on green innovation. R&D investment is measured by the amount of expenditure invested in R&D activities in the current year, and government subsidies are measured by the total amount of government subsidies received by enterprises in the current year, while the income tax burden is measured by the actual income tax rate of the enterprise.

#### 4.2.4. Control Variables

Drawing on the research of Peng and Tao (2022) [11] and Zhai et al. (2022) [18], this paper selects enterprise size, establishment time, financial leverage, growth ability, profitability, board size, proportion of independent directors, enterprise value, equity concentration, and the integration of two functions as control variables.

The design of the main variables is shown in Table 1 below.

### 4.3. Empirical Model Design

First, in order to test the impact of enterprise digital transformation on green innovation, this paper constructs the following fixed effect model:

(1) $$GI_{i,t+1}=\alpha_{0}+\alpha_{1}DT_{i,t}+\alpha_{3}Controls_{i,t}+year_{t}+ind_{i}+\varepsilon_{i,t}$$

Second, in order to test the transmission mechanism of R&D investment, government subsidies, and income tax burden on the impact of digital transformation on green innovation, this paper constructs the following fixed effect models:

(2) $$Mediator_{i,t}=\beta_{0}+\beta_{1}DT_{i,t}+\beta_{2}Controls_{i,t}+year_{t}+ind_{i}+\varepsilon_{i,t}$$

(3) $$GI_{i,t+1}=\gamma_{0}+\gamma_{1}Mediator_{i,t}+\gamma_{2}Controls_{i,t}+year_{t}+ind_{i}+\varepsilon_{i,t}$$

(4) $$GI_{i,t+1}=\mu_{0}+\mu_{1}Mediator_{i,t}+\mu_{2}DT_{i,t}+\mu_{3}Controls_{i,t}+year_{t}+ind_{i}+\varepsilon_{i,t}$$

In models (1)–(4), $GI_{i,t+1}$ represents green innovation, and $DT_{i,t}$ represents the degree of digital transformation of enterprises, including the overall digital transformation degree, the underlying technology architecture, and the technology practice application. $Mediator_{i,t}$ represents the mediating variables, including R&D investment, government subsidies, and income tax burden. $Controls_{i,t}$ represents all control variables, *α*_0_, *β*_0_, *γ*_0_, and *μ*_0_ are the intercept items, *α*_1~*n*_, *β*_1~*n*_, *γ*_1~*n*_, and *μ*_1~*n*_ are the correlation coefficients, $year_{t}$ represents the time fixed effect, $ind_{i}$ represents the industry fixed effect, and $\varepsilon_{i,t}$ is a random error term.

### 4.4. Descriptive Statistics

The descriptive statistical results of the main variables are presented in Table 2. The maximum value of enterprise green innovation is 7.0851, and the minimum value is 0, indicating that the level of green innovation among enterprises varies significantly. The maximum value of the overall digital transformation degree (ODT) is 6.0544, the minimum value is 0.6931, the maximum value of the underlying technology architecture (UTA) is 5.6384, the minimum value is 0, the maximum value of the technology practice application (TPA) is 5.8889, and the minimum value is 0, indicating that the differences in the digital transformation degree between different enterprises are obvious. In addition, the variables such as enterprise size (SCA), profitability (ROA) and growth ability (GRO) have different degrees of differences in the sample interval, which can be better adapted to the empirical analysis of this paper.

## 5. Empirical Results

### 5.1. Empirical Analysis of the Impact of Enterprise Digital Transformation on Green Innovation

The empirical results of the impact of enterprise digital transformation on green innovation are presented in Table 3. Column (1) shows that the influence coefficient of overall digital transformation degree on green innovation is 0.0975, which is significant at the 1% level, demonstrating that digital transformation can enhance green innovation. Columns (2)–(3) provide a further distinction between the empirical results of the underlying technology architecture and the technology practice application. The influence coefficient of underlying technology architecture on green innovation is 0.1205, and the influence coefficient of technology practice application on green innovation is 0.0383, both of which are significant at the 1% level. This indicates that both the digital transformation at the application level and the digital transformation at the technology level have a significant positive impact on green innovation, which verifies Hypothesis 1. The regression results show that the greater the degree of enterprise digital transformation is, the more conducive it is to the improvement of green innovation level.

### 5.2. Transmission Mechanism Test

In order to clarify the transmission mechanism of the impact of digital transformation on enterprise green innovation, this paper conducts mediating effect tests from three aspects: R&D investment, government subsidies, and income tax burden.

#### 5.2.1. Transmission Mechanism Test of R&D Investment

Table 4 presents the empirical results of the mediating effect of R&D investment on the relationship between digital transformation and green innovation. Columns (1)–(3) show that the influence coefficient of overall digital transformation degree on R&D investment is 0.0024, the influence coefficient of underlying technology architecture on R&D investment is 0.0092, and the influence coefficient of technology practice application on R&D investment is 0.0068, all of which are significant at the 1% level. This shows that the higher the degree of digital transformation is, the larger the scale of R&D investment. It can be seen from Column (4) that the coefficient influence of R&D investment on green innovation is 2.2821, which is significant at the 1% level, indicating that the larger the scale of R&D investment is, the more output there is of enterprise green innovation.

The regression results of Model 4, after adding the mediating variable of R&D investment, are shown in Columns (5)–(7). It can be seen that the influence coefficients of overall digital transformation degree, underlying technology architecture, and technology practice application on green innovation are 0.0859, 0.0995, and 0.0365, respectively, which are all significant at the 1% level. By comparing the results in Table 3, it can be seen that the influence coefficient of digital transformation on green innovation decreases after the mediating variable is added, which proves that R&D investment plays a partial mediating effect between digital transformation and green innovation. This demonstrates that enterprises can increase R&D investment to boost green innovation output after digital transformation.

#### 5.2.2. Transmission Mechanism Test of Government Subsidies

Table 5 displays the empirical results of the mediating effect of government subsidies on the relationship between digital transformation and green innovation. The results in Columns (1)–(3) showing that the influence coefficient of overall digital transformation degree of enterprises on government subsidies is 0.0007 and the influence coefficient of underlying technology architecture on government subsidies is 0.0011, both of which are significant at the 1% level, while the technology practice application has no significant impact on government subsidies. This announces that in addition to technology practice application, the higher the degree of digital transformation of an enterprise, the more government subsidies it will receive. It can be seen from Column (4) that the influence coefficient of government subsidies on green innovation is 9.0323, which is significant at the 1% level, indicating that government subsidies obtained by enterprises are conducive to alleviating financing constraints on green innovation of enterprises, thereby promoting their green innovation output.

The regression results of Model 4, after adding the mediating variable of government subsidies, are presented in Columns (5)–(7). It can be seen that the influence coefficients of overall digital transformation degree, underlying technology architecture, and technology practice application on green innovation are 0.0875, 0.1087, and 0.0346, respectively, which are entirely significant at the 1% level. By comparing the results in Table 3, it can be seen that the influence coefficient of digital transformation on green innovation is reduced after the government subsidies variable is added, which demonstrates that government subsidies play a partial mediating effect between digital transformation and green innovation. Specifically, the green innovation output is upgraded by rising government subsidies after the digital transformation of enterprises.

#### 5.2.3. Transmission Mechanism Test of Income Tax Burden

The empirical results of the mediating effect of income tax burden on the relationship between digital transformation and green innovation are shown in Table 6. It is manifest from Columns (1)–(3) that the influence coefficient of overall digital transformation degree of enterprises on the income tax burden is −0.0034, and the influence coefficient of underlying technology architecture on the income tax burden is −0.0078, both of which are significant at the 1% level. This reveals that the higher the degree of digital transformation is, the lower the income tax burden of the enterprise. This may be attributable to the high degree of digital transformation of high-tech enterprises, which has benefited from China’s low tax rate on high-tech enterprises in recent years, reducing their entire income tax burden. Nevertheless, the influence coefficient of technology practice application on income tax burden is 0.0011 and not significant, which is probably because that technology may be affected by different external and internal environmental factors in the process of practical application. Column (4) proves that the influence coefficient of income tax burden on green innovation is −0.3627, which is significant at the 1% level, indicating that the enterprise income tax burden has a negative impact on green innovation output. Specifically, the lower the income tax burden is, the lighter the tax burden of the enterprise, in order that more funds will be invested in the field of green R&D and promote the innovation output.

The regression results of Model 4, after adding the mediating variable of income tax burden, are shown in Columns (5)–(7). It can be seen that the influence coefficients of overall digital transformation degree, underlying technology architecture, and technology practice application on green innovation are 0.0968, 0.1187, and 0.0388, respectively, which are all significant at the 1% level. It manifests that the impact coefficient of digital transformation on green innovation decreases slightly after adding the variable of income tax burden, by comparing the results of Table 3, which illustrates that the income tax burden plays a part of the intermediary effect between digital transformation and green innovation. This means that the output of green innovation is boosted by reducing the income tax burden after enterprise digital transformation.

### 5.3. Expansionary Analysis

In order to further test the impact of digital transformation on the quality of green innovation and the difference of the impact on green innovation of different types of enterprises and to provide specific and operable suggestions for promoting the development of green innovation of enterprises, this paper conducts empirical tests in groups according to the quality of green innovation, the nature of enterprise property rights, and whether it is a high-tech enterprise.

#### 5.3.1. Group Inspection According to the Quality of Green Innovation

Referring to the practice of Yu et al. (2019) [88], this paper adopts green invention patents to measure high-quality green innovation and green utility model patents to measure low-quality green innovation. The empirical test results of the impact of enterprise digital transformation on high-quality and low-quality green innovation are exhibited in Table 7. From Columns (1)–(3), it can be seen that the influence coefficients of overall digital transformation degree, underlying technology architecture, and technology practice application on high-quality green innovation are 0.0903, 0.1079, and 0.0400, respectively, and all of them are significant at the 1% level.

Columns (4)–(6) show that the influence coefficients of overall digital transformation degree and underlying technology architecture on low-quality green innovation are 0.0281 and 0.0423, both of which are significant at the 1% level. Nevertheless, the influence coefficient of technology practice application on low-quality green innovation is 0.0025 and insignificant. This manifests that the application of technology in practice cannot improve low-quality green innovation. The reason may be that the digital transformation at the application level of technology practice mainly focuses on the application and promotion of digital technology. Consequently, technology practice application has a significant impact on green invention patents that can improve the technological level of enterprises, while it has no significant impact on green utility model patents that change the shape and structure of environmental treatment products. The above results comprehensively reveal that the practical application of digital transformation technology is more conducive to promoting high-quality green innovation, and Hypothesis 2 is verified.

#### 5.3.2. Group Inspection According to the Nature of Property Rights

The empirical test results of the impact of enterprise digital transformation on the green innovation of state-owned enterprises and non-state-owned enterprises are shown in Table 8. As can be seen from Columns (1)–(3), the influence coefficients of overall digital transformation degree, underlying technology architecture, and technology practice application on green innovation of state-owned enterprises are 0.1405, 0.1561, and 0.0747, respectively, and all of them are significant at the 1% level. According to Columns (4)–(6), the influence coefficients of the above three independent variables on green innovation of non-state-owned enterprises are 0.0242, 0.0390, and 0.0266, respectively, which are all significant at the 1% level. It can be seen that the influence coefficient of the degree of digital transformation on the green innovation of non-state-owned enterprises is significantly smaller than that of state-owned enterprises, which indicates that digital transformation is more conducive to increasing the green innovation output of state-owned enterprises. Hypothesis 3 is verified. It is demonstrated that state-owned enterprises can obtain more government subsidies in the case of digital transformation, to obtain more funds for green innovation activities, which increases their green innovation output.

#### 5.3.3. Group Inspection According to Whether High-Tech Enterprises or Not

The empirical test results of the impact of enterprise digital transformation on the green innovation of high-tech enterprises and non-high-tech enterprises are shown in Table 9. It can be seen from Columns (1)–(3) that the influence coefficients of overall digital transformation degree, underlying technology architecture, and technology practice application on green innovation of high-tech enterprises are 0.1067, 0.1167, and 0.0454, respectively, and all of them are significant at the 1% level, indicating that digital transformation significantly promotes the green innovation of high-tech enterprises. Nevertheless, the above three independent variables have no significant impact on the green innovation of non-high-tech enterprises, according to Columns (4)–(6). It can be seen that the degree of digital transformation has a more significant impact on the green innovation of high-tech enterprises compared with non-high-tech enterprises, which illustrates that digital transformation is more conducive to increasing the green innovation output of high-tech enterprises. Thus, Hypothesis 4 is verified. The above results comprehensively indicate that high-tech enterprises can obtain more tax incentives with the help of resource advantages, thereby reducing the cost of green R&D and promoting the output of green innovation.

### 5.4. Robustness Test

The above research results demonstrate that digital transformation has enhanced enterprise green innovation significantly. In order to test the robustness of these results, a robustness test is conducted using green patent authorizations (GIA) with a lag of one period, green patent applications with a lag of two periods (GI-2), alternative independent variables, and other testing methods.

#### 5.4.1. Green Patent Authorization Quantity with a Lag of One Period

In relation to the applications of green patents, the authorizations of green patents have been reviewed more rigorously by intellectual property management agencies, indicating that enterprises have a higher level of green innovation. Accordingly, green patent authorizations are selected as the alternative variable for robustness test. Table 10 exhibits the empirical results of the robustness test using the number of green patent authorizations. It can be seen that the influence coefficients of overall digital transformation degree, underlying technology architecture, and technology practice application on the number of green innovation authorizations are all significant at the 1% level, which signifies digital transformation has a remarkable role in promoting enterprise green innovation. This indicates that the conclusions of this paper are robust.

#### 5.4.2. Green Patent Applications with a Lag of Two Periods

In order to further test the robustness of the impact of enterprise digital transformation on green innovation, the number of green patent applications with a lag of two periods is used to carry out the robustness test. Results of the robustness test using the number of green patent applications with a lag of two periods are displayed in Table 11. It shows that the influence coefficients of overall digital transformation degree, underlying technology architecture, and technology practice application on green innovation are all significant at the 1% level, indicating that digital transformation significantly promotes enterprise green innovation, which proves the robustness of the conclusions of this paper again.

#### 5.4.3. Test Results after Re-Measurement Based on Digital Transformation

Furthermore, this paper adopts the method of replacing independent variables to carry out the robustness test. The independent variables are respectively replaced by whether the enterprise is digital transformation (DTN), the proportion of digital intangible assets to intangible assets (DIA), the proportion of text times in the overall digital transformation level to the number of words in the annual report (PODT), the proportion of text times in the underlying technology architecture level to the number of words in the annual report (PUTA), and the proportion of text times in the technology practice application level to the number of words in the annual report (PTPA). The empirical results of the impact of enterprise digital transformation on its green innovation, after re-measuring the digital transformation, are shown in Table 12. The research results demonstrate that the above-mentioned alternative variables of enterprise digital transformation are significantly and positively related to enterprise green innovation. This reveals that the digital transformation of enterprises can significantly promote the green innovation of enterprises and further verifies the robustness of the conclusions of this paper.

#### 5.4.4. Test Results of Negative Binomial Regression Model

Since the value of enterprise green innovation application (GIS) is a non-negative integer and its variance is greater than the expected value, the negative binomial regression model is applicable. Therefore, this paper also uses negative binomial regression model to test the robustness of the impact of digital transformation on enterprise green innovation. The empirical results in Table 13 disclose that the three core independent variables, the overall digital transformation degree, the underlying technology architecture, and the technology practice application, are still positively correlated with enterprise green innovation at the significance level of 1%, which validates the conclusion that enterprise digital transformation significantly promotes enterprise green innovation again.

### 5.5. Endogenous Problem

In order to solve the endogenous problem that may be caused by missing variables or reverse causality, this paper uses the instrumental variable method to test the reliability of the results. Referring to the practices of Nunn and Qian (2014) [102] Zhou et al. (2021) [103], and Sun et al. (2022) [104], the logarithm of the multiplication term (GD) of the number of Chinese Internet users in the previous year and the number of fixed telephones per 10,000 people in 1984 as the instrumental variable is selected for the empirical test. The empirical results in Table 14 manifest that the influence coefficients of the second stage of digital transformation on green innovation are still significantly positive after adding the instrumental variable, indicating that the main conclusions of this paper remain consistent.

## 6. Discussion

Nevertheless, this research still has some limitations. First of all, digital transformation is a new business model that covers information technology, organizational structure, corporate culture, and other elements, including both business digital transformation and strategic digital transformation. Consequently, how to accurately measure digital transformation has become one of the hot topics discussed by scholars. The existing research mainly adopts the text mining method to measure digital transformation, and this paper also uses this method for reference, while the deficiency is that the application of digital transformation technology at the operational level of enterprises is not reflected. Hence, how to accurately measure the variable of digital transformation, so as to give prominence to the application of digital technology in practice, is one of the future research directions of this research.

There is one more point, the scope of green innovation includes input, output, transformation, etc. This paper uses green patent output to measure green innovation, while it does not discuss the impact of digital transformation on green innovation investment and achievement transformation extensively. Accordingly, whether digital transformation will affect the green innovation investment and achievement transformation income of enterprises is also one of our important research fields in the future.

## 7. Conclusions 

This paper explores the relationship between digital transformation and enterprise green innovation and the transmission mechanism. Using the data of Chinese A-share listed companies from 2010 to 2020, this paper tests the impact of digital transformation on enterprise green innovation by structuring the time and industry dual fixed effect models. The specific conclusions are as follows: First, the underlying technology architecture, the technology practice application, and the overall digital transformation degree have significantly promoted the green innovation of enterprises, which provides new evidence for exploring the impact of digital transformation on the green innovation of micro enterprises from the overall perspective. Second, digital transformation can increase the output of green innovation by increasing R&D investment, amplifying government subsidies, and reducing the income tax burden, which verifies the transmission mechanism of the impact of digital transformation on green innovation from the perspective of resource-based theory and dynamic capacity theory. Third, the results of expansionary analysis demonstrate that the application of technology practice in digital transformation has a more significant impact on high-quality green innovation, and digital transformation is more conducive to promoting green innovation of high-tech enterprises and state-owned enterprises, which provide guidance for the reform of government green innovation policies and the practice of enterprise green innovation activities.

From the perspective of China’s current digital economy strategy and enterprise digital transformation practice, improving the vitality and quality of enterprise green innovation is a pivotal measure to promote sustainable economic development and green transformation development. Accordingly, from the perspective of theoretical contribution, this research systematically combs the latest literature related to digital transformation and green innovation and analyzes the impact mechanism of digital transformation on enterprise green innovation from the perspectives of dynamic capability theory and resource-based theory, expanding the research field of digital transformation and enriching the research on the influencing factors of green innovation. Furthermore, from the perspective of practical contributions, this research can provide policy suggestions for the government to improve the green innovation level of enterprises by promoting the construction of digital infrastructure, increasing financial subsidies and tax incentives, and boosting the green development of regional economy, thus contributing to the realization of the sustainable development goals of “carbon peak” and “carbon neutral”. There is one more point: this research can also provide reference for digital transformation enterprises to accelerate green innovation by increasing R&D investment, obtaining government subsidies, and tax policy support, thus helping enterprises to achieve green and sustainable development through digital transformation.

## 8. Recommendations

Based on the above research results, the following recommendations are proposed from the perspective of the government and enterprises.

Recommendations to the government. First, the government should strengthen the infrastructure construction of enterprise digital transformation. The government should further consolidate the infrastructure hardware and network system construction required by the digital transformation of enterprises such as 5G base stations, cloud computing platforms, and big data centers, expand the coverage of digital infrastructure, and provide strong support for enterprises to apply data processing technology, implement digital simulation, and implement digital transformation. Second, the government should promote high-quality green technology innovation by providing government subsidies and preferential tax policies. It has been proven that digital transformation has a more significant impact on high-quality green innovation, and government subsidies and income tax burden play an intermediary role. Combined with these research conclusions, the government should reduce the R&D cost of enterprises’ digital transformation, ease the financing constraints of green R&D input, and encourage enterprises to fulfil high-quality green innovation by increasing government subsidies and tax incentives. Third, the government should select high-tech enterprises and state-owned enterprises to carry out a digital transformation pilot. Combined with the research conclusions that digital transformation has a more significant impact on the green innovation of high-tech and state-owned enterprises, the government should select typical high-tech and state-owned enterprises to establish digital-transformation-innovation pilot zones, give play to the advantages of green technology resources and dynamic capabilities of enterprises, output high-quality green innovation achievements to provide demonstration for other enterprises, and accelerate other enterprises to accomplish digital transformation and improve their green sustainable development ability.

Recommendations to enterprises. First, enterprises should increase R&D investment to maintain dynamic capabilities, while implementing digital transformation, focus on the high-quality green environmental protection technology and green product R&D, and increase high-quality green innovation output. Second, enterprises should make use of the advantages of digital technology resources to win the support of government subsidies and preferential tax policies, reduce financing constraints, and boost the improvement of green innovation ability. Third, high-tech enterprises and state-owned enterprises should take advantage of the dynamic capabilities and innovation resources, enhance their green innovation capabilities through digital transformation, and promote their digital transformation and green sustainable development. Other enterprises should also follow the development trend of digital transformation, make full use of the mature cloud platform established by digital service providers to achieve digital transformation.

## Figures and Tables

**Table 1:** Definition of main variables.

Variable Type	Variable Name		Variable Symbol	Variable Interpretation
Dependent variables	Green innovation	Total green innovation	GI	The number of green patent applications lagging one year added to 1 to take the natural logarithm
		High-quality green innovation	HGI	The number of green invention patent applications lagging one year added to 1 to take the natural logarithm
		Low-quality green innovation	LGI	The number of green utility model patent applications lagging one year added to 1 to take the natural logarithm
Independent variables	Digital transformation	Overall digital transformation degree	ODT	The total number of texts in the five dimensions of artificial intelligence, big data, cloud computing, blockchain and digital technology application added to 1 to take the natural logarithm
		Underlying technology architecture	UTA	The total number of texts in the four dimensions of artificial intelligence, big data, cloud computing and blockchain added to 1 to take the natural logarithm
		Technology practice application	TPA	The number of texts in the digital technology application added to 1 to take the natural logarithm
Mediating variables	R&D investment		INP	R&D investment/operating income
	Government subsidies		SUB	Amount of government subsidies/total assets
	Income tax burden		TAX	Total income tax expense/profit
Control variables	Enterprise size		SCA	Natural logarithm of total assets of the enterprise
	Establishment time		TIM	Natural logarithm of enterprise establishment time
	Growth ability		GRO	Increase in operating income/opening operating income
	Financial leverage		LEV	Total liabilities/total assets at the end of the period
	Profitability		ROA	Net profit/total assets
	Enterprise value		TQ	Tobin’ Q value
	Equity concentration		STO	Natural logarithm of shareholding ratio of top 10 shareholders
	Board size		BOA	Natural logarithm of the number of directors
	Proportion of independent directors		RID	Proportion of independent directors in all directors
	Integration of two functions		DUA	If the chairman and CEO are held by the same individual, it is 1, otherwise it is 0

**Table 2:** Descriptive statistics of main variables.

Variable	Obs	Mean	Std.Dev.	Min	Max
GI	11,693	0.4654	0.9019	0.0000	7.0851
ODT	11,693	2.0450	1.1386	0.6931	6.0544
UTA	11,693	1.1259	1.2188	0.0000	5.6384
TPA	11,693	1.5065	1.1014	0.0000	5.8889
SCA	11,693	22.1792	1.2893	17.8132	28.5085
TIM	11,693	2.8267	0.3628	1.0986	4.1271
GRO	11,693	0.4330	1.0961	−0.7891	10.2879
LEV	11,693	0.4100	0.2030	0.0493	0.9796
ROA	11,693	0.0373	0.0690	−0.3657	0.2062
TQ	11,693	1.0854	0.3506	0.5343	4.6389
STO	11,693	4.0585	0.2787	2.3001	4.6265
BOA	11,693	2.2346	0.1786	1.6094	2.9444
RID	11,693	0.3777	0.0568	0.1818	0.8000
DUA	11,693	0.3095	0.4623	0.0000	1.0000

**Table 3:** Empirical results of digital transformation and green innovation.

Variable	(1)	(2)	(3)
	Model 1		
	GI	GI	GI
ODT	0.0975 ***		
	−11.6143		
UTA		0.1205 ***	
		−15.0651	
TPA			0.0383 ***
			−4.823
SCA	0.1611***	0.1623 ***	0.1678 ***
	−12.8567	−13.0593	−13.257
TIM	−0.1211 ***	−0.1165 ***	−0.1330 ***
	(−4.8009)	(−4.6010)	(−5.2638)
GRO	−0.0037	−0.0067	−0.0002
	(−0.6442)	(−1.1960)	(−0.0274)
LEV	0.3296 ***	0.3219 ***	0.3101***
	−6.7944	−6.691	−6.372
ROA	1.1129 ***	1.0923 ***	1.1038 ***
	−10.4702	−10.2916	−10.3866
TQ	0.1072 ***	0.1048 ***	0.1239 ***
	−3.8042	−3.7586	−4.356
STO	−0.0667 **	−0.0446	−0.0850 ***
	(−2.2507)	(−1.5074)	(−2.8424)
BOA	0.05	0.0686	0.0332
	−0.7562	−1.0503	−0.4964
RID	−0.2614	−0.2459	−0.257
	(−1.4688)	(−1.4025)	(−1.4321)
DUA	0.0375 **	0.0393 **	0.0441 **
	−2.0534	−2.1674	−2.4047
CONS	−3.1735 ***	−3.2311 ***	−3.1318 ***
	(−8.9686)	(−9.2179)	(−8.7899)
year	YES	YES	YES
ind	YES	YES	YES
N	11,693	11,693	11,693
R^2^	0.1342	0.1424	0.1248

*t* statistics in parentheses; ** *p* < 0.05, *** *p* < 0.01.

**Table 4:** Test results of transmission mechanism of R&D investment.

Variable	(1)	(2)	(3)	(4)	(5)	(6)	(7)
	Model 2			Model 3	Model 4		
	INP	INP	INP	GI	GI	GI	GI
ODT	0.0068 ***				0.0859 ***		
	(11.4721)				(9.1276)		
UTA		0.0092 ***				0.0995 ***	
		(16.7464)				(11.3331)	
TPA			0.0024 ***				0.0365 ***
			(4.0595)				(4.0871)
INP				2.2821 ***	2.0607 ***	1.8752 ***	2.2466 ***
				(11.4424)	(10.6980)	(9.9605)	(11.3241)
SCA	0.0017 ***	0.0017 ***	0.0022 ***	0.1630 ***	0.1542 ***	0.1563 ***	0.1591 ***
	(2.7643)	(2.8150)	(3.5295)	(11.4857)	(10.9287)	(11.1479)	(11.1806)
TIM	−0.0162 ***	−0.0158 ***	−0.0172 ***	−0.0907 ***	−0.0790 **	−0.0796 ***	−0.0870 ***
	(−8.5705)	(−8.3929)	(−8.9886)	(−2.9513)	(−2.5736)	(−2.5922)	(−2.8305)
GRO	0.0024 ***	0.0022 ***	0.0027 ***	−0.0052	−0.0092	−0.0109	−0.0061
	(4.5725)	(4.1898)	(5.0032)	(−0.6641)	(−1.1953)	(−1.4349)	(−0.7851)
LEV	−0.0699 ***	−0.0699 ***	−0.0710 ***	0.5811 ***	0.5820 ***	0.5670 ***	0.5816 ***
	(−16.2383)	(−16.2751)	(−16.3078)	(9.8371)	(9.8962)	(9.6855)	(9.8467)
ROA	−0.1020 ***	−0.1031 ***	−0.1025 ***	1.4572 ***	1.4532 ***	1.4192 ***	1.4689 ***
	(−7.7619)	(−7.9232)	(−7.7127)	(12.2508)	(12.2204)	(11.9668)	(12.3222)
TQ	0.0334 ***	0.0329 ***	0.0347 ***	0.0383	0.0237	0.0274	0.0316
	(10.9723)	(10.7569)	(11.2875)	(1.1143)	(0.6964)	(0.8121)	(0.9198)
STO	−0.0148 ***	−0.0127 ***	−0.0166 ***	−0.0422	−0.0197	−0.0038	−0.0387
	(−5.7780)	(−4.9761)	(−6.3965)	(−1.1750)	(−0.5526)	(−0.1077)	(−1.0788)
BOA	0.0022	0.0035	0.0010	0.0438	0.0578	0.0703	0.0432
	(0.5272)	(0.8495)	(0.2492)	(0.5491)	(0.7281)	(0.8911)	(0.5408)
RID	0.0169	0.0180	0.0172	−0.1847	−0.1898	−0.1735	−0.1896
	(1.3802)	(1.4934)	(1.3935)	(−0.8578)	(−0.8838)	(−0.8166)	(−0.8772)
DUA	0.0060 ***	0.0060 ***	0.0066 ***	0.0415 **	0.0323	0.0346 *	0.0375 *
	(4.9197)	(4.9224)	(5.3520)	(2.0189)	(1.5770)	(1.6961)	(1.8273)
CONS	0.0578 ***	0.0510 **	0.0644 ***	−3.2296 ***	−3.2632 ***	−3.3190 ***	−3.1850 ***
	(2.7864)	(2.4878)	(3.0527)	(−7.9981)	(−8.1013)	(−8.2891)	(−7.8621)
year	YES	YES	YES	YES	YES	YES	YES
ind	YES	YES	YES	YES	YES	YES	YES
N	9113	9113	9113	9113	9113	9113	9113
R^2^	0.2987	0.3129	0.2878	0.1248	0.1332	0.1378	0.1265

*t* statistics in parentheses; * *p* < 0.1, ** *p* < 0.05, *** *p* < 0.01.

**Table 5:** Test results of transmission mechanism of government subsidies.

Variable	(1)	(2)	(3)	(4)	(5)	(6)	(7)
	Model 2			Model 3	Model 4		
	SUB	SUB	SUB	GI	GI	GI	GI
ODT	0.0007 ***				0.0875 ***		
	(8.7349)				(10.4894)		
UTA		0.0011 ***				0.1087 ***	
		(13.7841)				(13.5655)	
TPA			0.0001				0.0346 ***
			(0.8205)				(4.3807)
SUB				9.0323 ***	8.2899 ***	7.3534 ***	9.0024 ***
				(6.1197)	(5.8823)	(5.4193)	(6.1429)
SCA	−0.0007 ***	−0.0007 ***	−0.0006 ***	0.1785 ***	0.1684 ***	0.1691 ***	0.1747 ***
	(−7.3924)	(−7.5280)	(−6.6625)	(13.8408)	(13.1301)	(13.2847)	(13.5183)
TIM	−0.0011 ***	−0.0010 ***	−0.0012 ***	−0.1176 ***	−0.1046 ***	−0.1023 ***	−0.1141 ***
	(−4.2165)	(−3.9676)	(−4.5837)	(−4.5967)	(−4.1031)	(−4.0016)	(−4.4679)
GRO	0.0000	−0.0000	0.0001	0.0005	−0.0032	−0.0061	0.0000
	(0.4226)	(−0.1094)	(0.8046)	(0.0898)	(−0.5240)	(−1.0260)	(0.0006)
LEV	0.0018 ***	0.0018 ***	0.0016 ***	0.2865 ***	0.3108 ***	0.3042 ***	0.2933 ***
	(3.1812)	(3.1263)	(2.8734)	(5.7694)	(6.2711)	(6.1850)	(5.9013)
ROA	0.0138 ***	0.0137 ***	0.0137 ***	0.9807 ***	1.0135 ***	1.0072 ***	0.9962 ***
	(9.2284)	(9.1827)	(9.1357)	(9.0960)	(9.3604)	(9.3187)	(9.2070)
TQ	0.0029 ***	0.0028 ***	0.0031 ***	0.1099 ***	0.0866 ***	0.0855 ***	0.1019 ***
	(5.2950)	(5.1508)	(5.6324)	(3.7457)	(2.9728)	(2.9593)	(3.4680)
STO	−0.0003	−0.0000	−0.0004	−0.0759**	−0.0572*	−0.0370	−0.0739**
	(−0.9114)	(−0.0809)	(−1.3889)	(−2.5031)	(−1.9048)	(−1.2353)	(−2.4442)
BOA	0.0013 **	0.0016 **	0.0012 *	−0.0095	0.0066	0.0260	−0.0110
	(1.9641)	(2.2961)	(1.7831)	(−0.1416)	(0.0994)	(0.3955)	(−0.1647)
RID	0.0043 **	0.0044 **	0.0044 **	−0.3182 *	−0.3238 *	−0.3065 *	−0.3257 *
	(1.9614)	(2.0279)	(1.9808)	(−1.7534)	(−1.7940)	(−1.7195)	(−1.7897)
DUA	−0.0002	−0.0002	−0.0001	0.0487 ***	0.0387 **	0.0403 **	0.0448 **
	(−1.0139)	(−1.0508)	(−0.6407)	(2.6443)	(2.1111)	(2.2116)	(2.4335)
CONS	0.0199 ***	0.0194 ***	0.0200 ***	−3.3470 ***	−3.3256 ***	−3.3630 ***	−3.2962 ***
	(5.7538)	(5.6577)	(5.7621)	(−9.3022)	(−9.2597)	(−9.4393)	(−9.1287)
year	YES	YES	YES	YES	YES	YES	YES
ind	YES	YES	YES	YES	YES	YES	YES
N	11,461	11,461	11,461	11,461	11,461	11,461	11,461
R^2^	0.0979	0.1081	0.0927	0.1287	0.1377	0.1443	0.1302

*t* statistics in parentheses; * *p* < 0.1, ** *p* < 0.05, *** *p* < 0.01.

**Table 6:** Test results of transmission mechanism of income tax burden.

Variable	(1)	(2)	(3)	(4)	(5)	(6)	(7)
	Model 2			Model 3	Model 4		
	TAX	TAX	TAX	GI	GI	GI	GI
ODT	−0.0034 **				0.0968 ***		
	(−2.3253)				(11.5319)		
UTA		−0.0078 ***				0.1187 ***	
		(−5.7052)				(14.7987)	
TPA			0.0011				0.0388 ***
			(0.7565)				(4.8829)
TAX				−0.3627 ***	−0.3493 ***	−0.3193 ***	−0.3645 ***
				(−7.4850)	(−7.2710)	(−6.7118)	(−7.5282)
SCA	0.0009	0.0011	0.0004	0.1726 ***	0.1619 ***	0.1633 ***	0.1684 ***
	(0.5561)	(0.7105)	(0.2487)	(13.6197)	(12.8941)	(13.1042)	(13.2758)
TIM	0.0184 ***	0.0177 ***	0.0191 ***	−0.1316 ***	−0.1163 ***	−0.1126 ***	−0.1277 ***
	(4.1986)	(4.0359)	(4.3393)	(−5.2036)	(−4.6182)	(−4.4531)	(−5.0611)
GRO	0.0013	0.0016	0.0011	0.0012	−0.0029	−0.0058	0.0006
	(0.8117)	(1.0107)	(0.7094)	(0.2113)	(−0.5053)	(−1.0321)	(0.0950)
LEV	0.0283 ***	0.0280 ***	0.0294 ***	0.3117 ***	0.3381 ***	0.3285 ***	0.3197 ***
	(2.6135)	(2.5914)	(2.7199)	(6.3604)	(6.9217)	(6.7820)	(6.5180)
ROA	0.3648 ***	0.3653 ***	0.3662 ***	1.2161 ***	1.2380 ***	1.2069 ***	1.2336 ***
	(19.4762)	(19.4807)	(19.5407)	(11.2238)	(11.4090)	(11.1532)	(11.3507)
TQ	−0.0252 ***	−0.0242 ***	−0.0263 ***	0.1196 ***	0.0948 ***	0.0928 ***	0.1113 ***
	(−3.9734)	(−3.8325)	(−4.1515)	(4.2231)	(3.3711)	(3.3351)	(3.9237)
STO	−0.0032	−0.0053	−0.0025	−0.0858 ***	−0.0653 **	−0.0435	−0.0838 ***
	(−0.5512)	(−0.8988)	(−0.4206)	(−2.8542)	(−2.1968)	(−1.4675)	(−2.7948)
BOA	0.0052	0.0035	0.0056	0.0342	0.0491	0.0671	0.0321
	(0.4825)	(0.3280)	(0.5255)	(0.5149)	(0.7436)	(1.0289)	(0.4824)
RID	0.0688 **	0.0683 **	0.0680 **	−0.2244	−0.2402	−0.2276	−0.2343
	(2.4059)	(2.3904)	(2.3810)	(−1.2547)	(−1.3501)	(−1.2975)	(−1.3059)
DUA	−0.0094 ***	−0.0092 ***	−0.0099 ***	0.0446 **	0.0339 *	0.0363 **	0.0403 **
	(−3.0160)	(−2.9561)	(−3.1759)	(2.4324)	(1.8591)	(2.0011)	(2.1958)
CONS	0.0166	0.0201	0.0186	−3.1931 ***	−3.1814 ***	−3.2406 ***	−3.1352 ***
	(0.2964)	(0.3594)	(0.3315)	(−9.0001)	(−9.0050)	(−9.2525)	(−8.8144)
year	YES	YES	YES	YES	YES	YES	YES
ind	YES	YES	YES	YES	YES	YES	YES
N	11,666	11,666	11,666	11,666	11,666	11,666	11,666
R^2^	0.0916	0.0938	0.0912	0.1264	0.1374	0.1451	0.1283

*t* statistics in parentheses; * *p* < 0.1, ** *p* < 0.05, *** *p* < 0.01.

**Table 7:** Group test results according to green innovation quality.

Variable	(1)	(2)	(3)	(4)	(5)	(6)
	Model 1					
	HGI			LGI		
ODT	0.0903 ***			0.0281 ***		
	(12.2378)			(5.0223)		
UTA		0.1079 ***			0.0423 ***	
		(14.9829)			(8.2684)	
TPA			0.0400 ***			0.0025
			(5.8227)			(0.4685)
SCA	0.1603 ***	0.1617 ***	0.1660 ***	0.0883 ***	0.0881 ***	0.0912 ***
	(14.1398)	(14.3657)	(14.4610)	(9.5324)	(9.5557)	(9.8427)
TIM	−0.0725 ***	−0.0689 ***	−0.0831 ***	−0.0769 ***	−0.0743 ***	−0.0811 ***
	(−3.5233)	(−3.3342)	(−4.0371)	(−4.1049)	(−3.9547)	(−4.3303)
GRO	0.0019	−0.0007	0.0051	−0.0083 **	−0.0096 ***	−0.0071 **
	(0.3822)	(−0.1453)	(1.0175)	(−2.3425)	(−2.7309)	(−2.0159)
LEV	0.2141 ***	0.2064 ***	0.1970 ***	0.2394 ***	0.2384 ***	0.2321 ***
	(5.2081)	(5.0628)	(4.7674)	(7.1451)	(7.1399)	(6.9399)
ROA	0.8091 ***	0.7900 ***	0.8026 ***	0.6115 ***	0.6059 ***	0.6054 ***
	(9.0331)	(8.8494)	(8.9449)	(8.2270)	(8.1411)	(8.1626)
TQ	0.1364 ***	0.1350 ***	0.1509 ***	0.0280	0.0256	0.0346 *
	(5.6408)	(5.6558)	(6.1573)	(1.4288)	(1.3093)	(1.7659)
STO	−0.0763 ***	−0.0572 **	−0.0930 ***	0.0106	0.0196	0.0049
	(−2.9667)	(−2.2235)	(−3.5742)	(0.5286)	(0.9832)	(0.2431)
BOA	0.0463	0.0625	0.0304	0.0309	0.0383	0.0265
	(0.7972)	(1.0894)	(0.5181)	(0.6467)	(0.8060)	(0.5534)
RID	−0.1396	−0.1253	−0.1368	−0.0814	−0.0768	−0.0779
	(−0.9105)	(−0.8289)	(−0.8829)	(−0.6473)	(−0.6154)	(−0.6190)
DUA	0.0346 **	0.0366 **	0.0403 ***	0.0425 ***	0.0424 ***	0.0453 ***
	0.1603 ***	(2.3828)	(2.5927)	(3.1562)	(3.1614)	(3.3650)
CONS	−3.3488 ***	−3.4008 ***	−3.3034 ***	−1.8376 ***	−1.8568 ***	−1.8384 ***
	(−10.5718)	(−10.8351)	(−10.3526)	(−7.2438)	(−7.3491)	(−7.2265)
year	YES	YES	YES	YES	YES	YES
ind	YES	YES	YES	YES	YES	YES
N	11,693	11,693	11,693	11,693	11,693	11,693
R^2^	0.1260	0.1344	0.1155	0.1134	0.1164	0.1115

*t* statistics in parentheses; * *p* < 0.1, ** *p* < 0.05, *** *p* < 0.01.

**Table 8:** Group test results according to the nature of property rights.

Variable	(1)	(2)	(3)	(4)	(5)	(6)
	Model 1					
	State−Owned Enterprises			Non−State−Owned Enterprises		
ODT	0.1405 ***			0.0242 ***		
	(7.3524)			(3.8137)		
UTA		0.1561 ***			0.0390 ***	
		(8.1336)			(6.8612)	
TPA			0.0747 ***			0.0266 ***
			(4.1395)			(3.0292)
SCA	0.2253 ***	0.2276 ***	0.2360 ***	0.0720 ***	0.0721 ***	0.1371 ***
	(9.4246)	(9.5504)	(9.7019)	(6.5226)	(6.6038)	(8.9678)
TIM	−0.1411 **	−0.1179 **	−0.1521 ***	−0.0984 ***	−0.0964 ***	−0.1515 ***
	(−2.4498)	(−2.0201)	(−2.6248)	(−4.8189)	(−4.7114)	(−5.4454)
GRO	−0.0108	−0.0168 *	−0.0055	−0.0027	−0.0037	0.0049
	(−1.0475)	(−1.6696)	(−0.5147)	(−0.5933)	(−0.8244)	(0.7120)
LEV	0.0539	0.0551	0.0384	0.3295 ***	0.3298 ***	0.4260 ***
	(0.5317)	(0.5440)	(0.3762)	(8.6546)	(8.6829)	(7.7528)
ROA	1.0925 ***	1.1532 ***	1.0748 ***	0.6452 ***	0.6362 ***	1.1057 ***
	(3.3023)	(3.4969)	(3.2596)	(8.5787)	(8.4656)	(10.1580)
TQ	0.0859	0.0995	0.1310 **	0.0445 **	0.0420 *	0.1244 ***
	(1.3377)	(1.5766)	(1.9830)	(1.9853)	(1.8823)	(3.9027)
STO	−0.3375 ***	−0.3126 ***	−0.3718 ***	0.0537 **	0.0625 ***	0.0165
	(−5.4955)	(−5.0259)	(−5.9552)	(2.2392)	(2.6158)	(0.4832)
BOA	0.0607	0.1054	0.0128	0.0230	0.0277	0.0092
	(0.4663)	(0.8232)	(0.0963)	(0.4377)	(0.5277)	(0.1234)
RID	−0.6026 *	−0.5034	−0.6157 *	−0.0026	−0.0059	−0.1851
	(−1.8277)	(−1.5559)	(−1.8318)	(−0.0175)	(−0.0395)	(−0.8757)
DUA	0.0059	0.0195	0.0108	0.0316 **	0.0305 **	0.0494 ***
	(0.1011)	(0.3399)	(0.1863)	(2.3020)	(2.2275)	(2.5824)
CONS	−3.3971 ***	−3.6248 ***	−3.3105 ***	−1.6050 ***	−1.6184 ***	−2.7236 ***
	(−5.4911)	(−5.9450)	(−5.2903)	(−4.8495)	(−4.9146)	(−5.8588)
year	YES	YES	YES	YES	YES	YES
ind	YES	YES	YES	YES	YES	YES
N	3466	3466	3466	8227	8227	8227
R^2^	0.2101	0.2163	0.1991	0.1071	0.1103	0.1043

*t* statistics in parentheses; * *p* < 0.1, ** *p* < 0.05, *** *p* < 0.01.

**Table 9:** Group test results according to whether high-tech enterprises or not.

Variable	(1)	(2)	(3)	(4)	(5)	(6)
	Model 1					
	High-Tech Enterprises			Non-High-Tech Enterprises		
ODT	0.1067 ***			−0.0011		
	(10.2492)			(−0.1505)		
UTA		0.1167 ***			0.0084	
		(12.2025)			(1.2122)	
TPA			0.0454 ***			0.0086
			(4.5264)			(0.8789)
SCA	0.2246 ***	0.2284 ***	0.2341 ***	0.0741 ***	0.0734 ***	0.1295 ***
	(12.6803)	(13.0019)	(13.0855)	(6.1903)	(6.1360)	(7.8649)
TIM	−0.0495	−0.0478	−0.0656 *	−0.0336	−0.0351	−0.0488 *
	(−1.4768)	(−1.4257)	(−1.9570)	(−1.5653)	(−1.6214)	(−1.6523)
GRO	−0.0006	−0.0034	0.0049	−0.0061 **	−0.0064 **	−0.0086 *
	(−0.0672)	(−0.3722)	(0.5094)	(−2.1716)	(−2.2644)	(−1.8854)
LEV	0.5163 ***	0.4997 ***	0.4932 ***	−0.0061	−0.0061	−0.0531
	(7.9479)	(7.7411)	(7.5603)	(−0.1357)	(−0.1373)	(−0.8514)
ROA	1.3388 ***	1.3251 ***	1.3221 ***	0.0769	0.0768	0.0750
	(10.0900)	(10.0002)	(9.9744)	(0.6989)	(0.7040)	(0.4980)
TQ	0.1211 ***	0.1217 ***	0.1440 ***	0.0544 **	0.0533 **	0.0992 ***
	(3.2618)	(3.3169)	(3.8489)	(2.2206)	(2.1816)	(3.0593)
STO	−0.0086	0.0044	−0.0366	0.0274	0.0279	0.0374
	(−0.2122)	(0.1087)	(−0.8924)	(1.2298)	(1.2617)	(1.1851)
BOA	0.0199	0.0304	−0.0022	0.1510 **	0.1526 **	0.1643 **
	(0.2265)	(0.3496)	(−0.0247)	(2.5349)	(2.5554)	(2.0899)
RID	−0.2503	−0.2297	−0.2340	0.0346	0.0353	0.0243
	(−1.0616)	(−0.9906)	(−0.9816)	(0.2316)	(0.2360)	(0.1105)
DUA	0.0347	0.0363	0.0390 *	0.0253	0.0241	0.0409 *
	(1.5535)	(1.6354)	(1.7371)	(1.4583)	(1.3922)	(1.7481)
CONS	−4.9154 ***	−4.9385 ***	−4.9006 ***	−1.9049 ***	−1.8912 ***	−3.1731 ***
	(−9.6972)	(−9.8390)	(−9.5923)	(−5.6991)	(−5.6719)	(−7.0616)
year	YES	YES	YES	YES	YES	YES
ind	YES	YES	YES	YES	YES	YES
N	8085	8085	8085	3608	3608	3608
R^2^	0.1417	0.1467	0.1321	0.1033	0.1036	0.1258

*t* statistics in parentheses; * *p* < 0.1, ** *p* < 0.05, *** *p* < 0.01.

**Table 10:** Robustness test results (1).

Variable	(1)	(2)	(3)
	Model 1		
	GIA	GIA	GIA
ODT	0.0576 ***		
	(8.0658)		
UTA		0.0762 ***	
		(11.4670)	
TPA			0.0172 **
			(2.5230)
SCA	0.1371 ***	0.1374 ***	0.1416 ***
	(12.1388)	(12.2614)	(12.4826)
TIM	−0.1270 ***	−0.1235 ***	−0.1346 ***
	(−5.6725)	(−5.4978)	(−6.0196)
GRO	−0.0012	−0.0032	0.0010
	(−0.2391)	(−0.6744)	(0.2086)
LEV	0.2810 ***	0.2772 ***	0.2684 ***
	(6.6766)	(6.6257)	(6.3743)
ROA	0.6643 ***	0.6524 ***	0.6567 ***
	(7.0211)	(6.9025)	(6.9342)
TQ	0.0272	0.0246	0.0382
	(1.1178)	(1.0175)	(1.5657)
STO	−0.0447 *	−0.0299	−0.0557 **
	(−1.7167)	(−1.1508)	(−2.1275)
BOA	0.0750	0.0874	0.0653
	(1.3182)	(1.5477)	(1.1427)
RID	−0.0104	−0.0012	−0.0064
	(−0.0670)	(−0.0077)	(−0.0413)
DUA	0.0527 ***	0.0534 ***	0.0572 ***
	(3.2242)	(3.2839)	(3.4932)
CONS	−2.6861 ***	−2.7219 ***	−2.6697 ***
	(−8.4893)	(−8.6561)	(−8.3973)
year	YES	YES	YES
ind	YES	YES	YES
N	11,693	11,693	11,693
R^2^	0.1317	0.1368	0.1270

*t* statistics in parentheses; * *p* < 0.1, ** *p* < 0.05, *** *p* < 0.01.

**Table 11:** Robustness test results (2).

Variable	(1)	(2)	(3)
	Model 1		
	GI−2	GI−2	GI−2
ODT	0.0988 ***		
	(10.1502)		
UTA		0.1238 ***	
		(13.2347)	
TPA			0.0346 ***
			(3.7510)
SCA	0.1816 ***	0.1844 ***	0.1888 ***
	(11.9825)	(12.2817)	(12.3465)
TIM	−0.1094 ***	−0.1065 ***	−0.1205 ***
	(−3.8930)	(−3.7761)	(−4.2741)
GRO	−0.0039	−0.0065	−0.0001
	(−0.6379)	(−1.1017)	(−0.0192)
LEV	0.2846 ***	0.2740 ***	0.2614 ***
	(4.9729)	(4.8291)	(4.5581)
ROA	1.2960 ***	1.2932 ***	1.2921 ***
	(9.1576)	(9.1209)	(9.1418)
TQ	0.1207 ***	0.1220 ***	0.1392 ***
	(3.5984)	(3.6837)	(4.1199)
STO	−0.0619 *	−0.0394	−0.0782 **
	(−1.7882)	(−1.1383)	(−2.2364)
BOA	0.0254	0.0434	0.0114
	(0.3384)	(0.5841)	(0.1506)
RID	−0.2420	−0.2319	−0.2291
	(−1.2116)	(−1.1767)	(−1.1381)
DUA	0.0354 *	0.0389 *	0.0412 *
	(1.6868)	(1.8676)	(1.9578)
CONS	−3.6096 ***	−3.7025 ***	−3.5947 ***
	(−8.5211)	(−8.8292)	(−8.4255)
year	YES	YES	YES
ind	YES	YES	YES
N	9304	9304	9304
R^2^	0.1363	0.1442	0.1272

*t* statistics in parentheses; * *p* < 0.1, ** *p* < 0.05, *** *p* < 0.01.

**Table 12:** Robustness test results (3).

Variable	(1)	(2)	(3)	(4)	(5)
	Model 1				
	GI	GI	GI	GI	GI
DTN	0.1467 ***				
	(13.1468)				
DIA		0.0932 ***			
		(4.3673)			
PODT			0.8303 ***		
			(7.5715)		
PUTA				1.6294 ***	
				(9.0404)	
PTPA					0.2693 **
					(2.0074)
SCA	0.1405 ***	0.1495 ***	0.1673 ***	0.1682 ***	0.1720 ***
	(17.6891)	(18.4430)	(13.1955)	(13.3548)	(13.5016)
TIM	−0.1411 ***	−0.1556 ***	−0.1285 ***	−0.1258 ***	−0.1382 ***
	(−9.1577)	(−9.9433)	(−5.0593)	(−4.9581)	(−5.4435)
GRO	0.0033	0.0026	−0.0018	−0.0029	0.0002
	(1.1749)	(0.8761)	(−0.3231)	(−0.5158)	(0.0347)
LEV	0.1911 ***	0.1864 ***	0.3108 ***	0.3073 ***	0.2988 ***
	(6.3526)	(6.0485)	(6.3919)	(6.3464)	(6.1364)
ROA	0.7531 ***	0.7678 ***	1.1024 ***	1.0834 ***	1.0920 ***
	(9.9267)	(9.9179)	(10.3677)	(10.2363)	(10.2631)
TQ	0.0719 ***	0.0731 ***	0.1142 ***	0.1204 ***	0.1289 ***
	(4.2383)	(4.1275)	(4.0239)	(4.2968)	(4.5016)
STO	−0.0510 ***	−0.0528 ***	−0.0711 **	−0.0588 **	−0.0889 ***
	(−2.7577)	(−2.7837)	(−2.3716)	(−1.9630)	(−2.9514)
BOA	0.1184 ***	0.1142 ***	0.0540	0.0647	0.0417
	(3.0310)	(2.8633)	(0.8106)	(0.9801)	(0.6221)
RID	−0.0662	−0.0454	−0.2008	−0.2025	−0.2125
	(−0.5729)	(−0.3854)	(−1.1230)	(−1.1409)	(−1.1829)
DUA	0.0255 **	0.0322 ***	0.0425 **	0.0465 **	0.0453 **
	(2.1942)	(2.7310)	(2.3168)	(2.5478)	(2.4642)
CONS	−2.8824 ***	−3.0221 ***	−3.1916 ***	−3.2807 ***	−3.1909 ***
	(−13.4442)	(−13.8180)	(−8.9819)	(−9.2805)	(−8.9378)
year	YES	YES	YES	YES	YES
ind	YES	YES	YES	YES	YES
N	25,114	24,727	11,634	11,634	11,634
R^2^	0.1191	0.1123	0.1292	0.1331	0.1235

*t* statistics in parentheses; ** *p* < 0.05, *** *p* < 0.01.

**Table 13:** Robustness test results (4).

Variable	(1)	(2)	(3)
	Model 1		
	GIS	GIS	GIS
ODT	0.2718 ***		
	(9.8825)		
UTA		0.3093 ***	
		(11.3531)	
TPA			0.1410 ***
			(5.3534)
SCA	0.6293 ***	0.6293 ***	0.6519 ***
	(19.2283)	(18.5792)	(20.3384)
TIM	−0.5495 ***	−0.5879 ***	−0.5484 ***
	(−5.3584)	(−5.7693)	(−5.4456)
GRO	0.0035	−0.0108	0.0291
	(0.1046)	(−0.3263)	(0.8783)
LEV	1.0898 ***	1.0902 ***	1.0424 ***
	(5.1681)	(5.1336)	(5.0387)
ROA	4.7607 ***	4.7841 ***	4.7529 ***
	(8.0875)	(7.9056)	(8.2989)
TQ	0.0715	0.0333	0.1374
	(0.5219)	(0.2286)	(1.1102)
STO	−0.0259	0.0389	−0.0931
	(−0.2306)	(0.3419)	(−0.8443)
BOA	−0.2892	−0.2630	−0.4441 **
	(−1.4275)	(−1.3058)	(−2.1896)
RID	−1.4449 **	−1.5380 **	−1.7708 ***
	(−2.3452)	(−2.4755)	(−2.8740)
DUA	0.2014 ***	0.2057 ***	0.2097 ***
	(3.0849)	(3.1008)	(3.2772)
CONS	−13.2860 ***	−13.0057 ***	−12.9556 ***
	(−13.0666)	(−12.7013)	(−12.9499)
year	YES	YES	YES
ind	YES	YES	YES
N	11,693	11,693	11,693

*z* statistics in parentheses; ** *p* < 0.05, *** *p* < 0.01.

**Table 14:** Test results of instrumental variable method.

Variable	First Stage			Second Stage		
	ODT	UTA	TPA	GI	GI	GI
ODT				2.1839 ***		
				(3.3206)		
UTA					2.2897 ***	
					(3.1844)	
TPA						2.0794 ***
						(3.2373)
GD	0.0531 ***	0.0507 ***	0.0558 ***			
	(3.4577)	(3.1952)	(3.5076)			
SCA	0.1137 ***	0.0820 ***	0.1111 ***	−0.0720	−0.0114	−0.0547
	(11.2335)	(7.5220)	(10.6969)	(−0.9344)	(−0.1813)	(−0.7389)
TIM	−0.1590 ***	−0.1657 ***	−0.0948 ***	0.2195 *	0.2517 *	0.0693
	(−5.3472)	(−5.1689)	(−3.1795)	(1.7358)	(1.7743)	(0.7569)
GRO	0.0432 ***	0.0599 ***	0.0176 **	−0.0933 ***	−0.1362 ***	−0.0356
	(4.7137)	(6.0466)	(2.0239)	(−2.6588)	(−2.8174)	(−1.6024)
LEV	−0.2882 ***	−0.1686 ***	−0.2086 ***	0.9155 ***	0.6720 ***	0.7198 ***
	(−4.7096)	(−2.5978)	(−3.2822)	(3.9864)	(3.5927)	(3.7612)
ROA	−0.2611	−0.0429	−0.4289 ***	1.6266 ***	1.1546 ***	1.9481 ***
	(−1.5749)	(−0.2368)	(−2.6315)	(4.0458)	(2.7686)	(4.3767)
TQ	0.2639 ***	0.2348 ***	0.2200 ***	−0.4299 **	−0.3911 **	−0.3111 *
	(7.7166)	(6.1883)	(6.1943)	(−2.3217)	(−2.1259)	(−1.9575)
STO	−0.2038 ***	−0.3480 ***	−0.0488	0.3584 **	0.7102 ***	0.0150
	(−5.7931)	(−9.2401)	(−1.3621)	(2.2859)	(2.6635)	(0.1780)
BOA	−0.1484 **	−0.2751 ***	0.0603	0.3701 **	0.6757 ***	−0.0795
	(−2.1934)	(−3.8369)	(0.8669)	(1.9948)	(2.5953)	(−0.4831)
RID	0.1686	0.0059	0.2803	−0.5742	−0.2195	−0.7889
	(0.8279)	(0.0272)	(1.3146)	(−1.2131)	(−0.4468)	(−1.5147)
DUA	0.1073 ***	0.0718 ***	0.1066 ***	−0.1919 **	−0.1220	−0.1792 **
	(5.0957)	(3.1982)	(4.9223)	(−2.2422)	(−1.6448)	(−2.1529)
CONS	−0.6662 *	−0.1290	−2.0241 ***	−2.8059 ***	−3.9654 ***	−0.0520
	(−1.9098)	(−0.3465)	(−5.6594)	(−3.7224)	(−4.7251)	(−0.0415)
year	Yes	Yes	Yes	Yes	Yes	Yes
ind	Yes	Yes	Yes	Yes	Yes	Yes
N	11,673	11,673	11,673	11,673	11,673	11,673

*t* statistics in parentheses; * *p* < 0.1, ** *p* < 0.05, *** *p* < 0.01.

## Data Availability

The data presented in this study are available on request from the corresponding author. The data are not publicly available due to privacy.
